# China's current situation and development of hospice and palliative care in critical care medicine

**DOI:** 10.3389/fmed.2023.1066006

**Published:** 2023-03-06

**Authors:** Longxiang Su, Xiaohong Ning

**Affiliations:** ^1^Department of Critical Care Medicine, State Key Laboratory of Complex Severe and Rare Diseases, Peking Union Medical College Hospital, Peking Union Medical College and Chinese Academy of Medical Sciences, Beijing, China; ^2^Department of Geriatric Medicine, State Key Laboratory of Complex Severe and Rare Diseases, Peking Union Medical College Hospital, Peking Union Medical College and Chinese Academy of Medical Sciences, Beijing, China

**Keywords:** critical care medicine, palliative care, hospice care, teaching, China

## 1. Introduction

Hospice and palliative care (HPC) is a novel inter-discipline with a history spanning the last 50 years, during which time, it has become an independent discipline in many countries. In 2000, the American Board of Hospice and Palliative Medicine stated that “palliative medicine is the medical specialty devoted to achieving the best possible quality of life of the patient and the family during the course of a life-threatening illness through the relief of suffering and the control of symptoms” (1). As a discipline focusing on the quality of life of patients with a finite life span, it provides hope in modern medicine, helping illuminate the dark moments of death and providing solutions to many difficult medical scenarios. However, in China, HPC has not yet become a specialized or sub-specialized subject, with a lack of corresponding education for most medical students/nursing students. Accordingly, there is a need to promote the dissemination and popularization of relevant knowledge of HPC in various ways.

## 2. Ignorance of the demand for HPC in critical care medicine

The therapeutic goal is a “cure” for patients with acute and critical illnesses receiving treatment in the hospital. However, despite a clear diagnosis of diseases with poor prognosis based on the use of assessment tools for prognosis, the need for, and practices of HPC are also seriously ignored in large hospitals guided by the mainstream values of “saving lives” and “resuscitation.” For example, according to a survey of two major acute care hospitals, one-third of the patients had HPC needs (2), suggesting an insufficiency of HPC care for these patients (3).

In critical care medicine, the topic related to end-of-life is a problem that cannot be ignored. ICU is an important place where the hospital provides life sustaining treatment for critically ill patients, so as to rebuild and maintain organ functions. The focus of attention in the ICU is “how to live.” It seems that talking about “death” means “giving up,” which means “medical failure.” Despite the continuous development of medical science and technology, the mortality rate in ICU is still as high as 20%−35% (4). The initial treatment provided to patients in ICU might cause more harm than good when patients start to develop organ failure or have no response to that treatment. Doctors in the ICU also have to consider how to cope with the imminent death of patients.

## 3. The definition of HPC

In 2000, the American Board of Hospice and Palliative Medicine stated that “palliative medicine is a medical specialty devoted to achieving the best possible quality of life for the patient and the family during the course of a life-threatening illness through the relief of suffering and the control of symptoms” (1). HPC is a discipline that can offer assistance to patients with a finite life span caused by serious diseases and their families. It can provide assistance to patients and their families, helping maintain the best possible quality of life by actively evaluating, discovering, and dealing with the patients' holistic suffering, including physical, psychological, spiritual, and social suffering so that they can live out the final stage of life smoothly. Significantly, HPC is available at any time according to the patient's needs, independent of the patient's age or the type of disease.

HPC is a subject that confronts the limitations of medical science and technology and is a subject that faces death. It can offer active assistance for patients and their families to understand the true meaning of life and death at ease. In other words, life has its limits. Death is not a failure of medicine but the law of life. Human beings cannot change this law. On the premise of conforming to this law, human beings should not only “fight” against disease but also timely reconcile with death. The word “timely” is the core and also the difficulty of HPC. Importantly, medicine has never been a technology based on theory only but a combination of science and humanity. Notably, practice in HPC is the most perfect demonstration of medical humanities.

## 4. Intimate association between critical care medicine and HPC

Seemingly, critical care medicine and HPC are two opposite disciplines. The former wants to “save life,” while the latter talks about “death.” In addition, in some people's opinion, critical care medicine is “technical medicine” and HPC is “talking medicine.”

Really? It will be apparent in a moment's consideration that the two disciplines are full of commonalities and intertwined so harmoniously. Both critical care medicine and HPC are concerned with the care of patients with serious diseases/life threats. The purpose of critical care medicine is to save lives, without excluding the maintenance of the quality of life and with the emphasis on coping with the topics of “death.” Simultaneously, HPC aims to maintain quality of life of patients, without excluding life extension and with emphasis spent talking about “how to live well.” Both critical care medicine and HPC share consistent values and goals to obtain a higher quality of life and human dignity while maintaining the life cycle. As mentioned in the definition of palliative care (PC) by WHO, all patients shall be provided with PC at the same time when receiving curative treatment. In clinical practice, there is no contradiction between saving/prolonging life and alleviating pain/maintaining the quality of life.

## 5. Indispensable roles of HPC in ICU

There are many problems to be coped with in ICU, such as patients' painful symptoms, family members' anxiety and depression, difficult decision-making, difficult communication, how to respect patients' autonomy, how to benefit patients rather than injuries, whether to withdraw or not to give a treatment, etc.

The incidence of painful symptoms in critically ill patients reached 27%−75% in ICU, and one-third of patients had delirium (5). In addition to alleviating the physical pain of patients, the medical team should also maintain their dignity and give them respect for life, which can be addressed to some extent by following the concept and practice of HPC. Clinically, besides the suffering of patients, their families are also suffering of which 57% have trauma-related pain and 70%−80% have depression (6).

In addition to coping with all types of suffering of patients and their families, medical staff in ICU also need to distinguish between “death” and “reversible deterioration” in the treatment process, which are generally difficult to discriminate. Medical staff will also have many psychological, moral, and ethical pressures under the aforementioned difficult situations. Medical staff who have not received adequate training for HPC are often powerless. In view of the above, it is important to carry out HPC-related assessments as well as early prediction and intervention for patients entering the ICU to reduce the suffering of many people.

Previous research has confirmed that the involvement of the HPC team can promote the early initiation of family meetings in the ICU, shorten the length of stay in the ICU, and the total length of stay in the hospital (7). As proposed by the latest guidelines for sepsis, it is suggested that PC should be taken into account in sepsis and septic shock according to the patient's situation (8). HPC Medical team in Peking Union Medical University Hospital, established in 2012, has begun and address the HPC of critically ill patients and has achieved good results.

## 6. The content of HPC in ICU

The core of HPC is to take care of critically ill patients in the ICU, control symptoms, and discuss the goals of care.

Part of the daily work of medical staff in the ICU is to comfort the patients receiving treatment in ICU physically, such as alleviating pain and improving symptoms such as thirst, anxiety, sleep disorders, and dyspnea. However, due to the limited energy of the medical staff and the therapeutic goal of “cure,” this part of the work may not be fully paid attention to and implemented in the clinical practice.

Discussing the goals of care is important for the treatment of critically ill patients. Meanwhile, the therapeutic goal should not be formulated by the medical team unilaterally but is the result of the joint decision-making of the multi-disciplinary team (MDT), the patient, and family members.

It is crucial that the goals of care are discussed with the patient themselves or the patient's family members. Following the principle of autonomy among the four basic principles of medical ethics, it is extremely important to respect the wishes of patients. Hence, patients are allowed to express their wishes and care goals. It is, however, not fully emphasized in China, and there is a long way to go compared with current international practices. The patient's serious illness is frequently not truthfully informed to the patient or their family, and even the medical staff regard this “white deception and lies” as “great love” and “filial piety.” Under the influence of the so-called love and filial piety, even if the patient himself/herself has decision-making ability and willingness, there is no opportunity for the patient to make decisions. In many cases, patients are delivered into ICU unwittingly, with corresponding autonomy completely disrespected. Advance care planning (ACP) is a method widely used abroad to express wishes. In China, there is also the text of “My Five Wishes” of the Living Will Promotion Association and the promotion based on the website, WeChat official accounts, and offline activities of the Association. If there is an opportunity, we can know the wishes of patients by communicating ACP or “My Five Wishes,” which, unfortunately, is extremely difficult to implement.

When patients do not have the opportunity to express their wishes, doctors generally communicate with their families about the therapeutic goals. There will be a great difference between the conclusions drawn by the families from the best interests of patients or their feelings when determining the goals. Some families may make some decisions based on the interests and wishes of the patients, e.g., “My father once said that he should not be intubated, he should go with dignity.” However, at present in China, more family members of patients make decisions from the perspective of “the common practice of the society,” “what others will think of me,” and “I don't want to leave any regrets.” Consequently, the wishes and feelings of patients are ignored to some extent. As reported by previous studies, in some cases, the family members were not really given such decision-making opportunities but were just informed by the medical team that had made decisions already.

Therefore, discussing the therapeutic goals is an important ability of doctors in the ICU. It is the routine work of the ICU and should also be regarded as an important part of the competency of doctors in the ICU. The content of HPC in the ICU should include discussing the therapeutic goals repeatedly at any time, providing patients with physical, psychological, social, and spiritual support, and family members with emotional and decision-making support, as well as self-pressure regulation of team members, in addition to the relief of physical symptoms. In the process of decision-making, it is the basic skill of doctors in the ICU to discuss with patients/families and team members not to give or withdraw a certain treatment.

## 7. Specific practice of HPC in ICU

It is a great challenge to help patients in the ICU get holistic care. Before the concept of emergence and intervention of HPC, we should make up our minds about the facts that life is limited and death may be coming, which is the biggest difficulty. In addition, it is also a great difficulty to balance the wishes of patients/families, doctors' therapeutic strategies, and patients' wishes.

From the perspective of international practice, there are two modes for ICU to practice HPC. The first is the integration mode (9), which allows medical staff in the ICU to learn the concept and knowledge of HPC, so as to promote a direct application of the concept of HPC to the specific practice such as the control of patients' painful symptoms, psychological, social, and spiritual support of patients, support of family members, family meetings, and joint decision-making. In other words, this mode allows the medical staff of critical care medicine to be responsible for the work of primary HPC. The other is consultation mode. HPC professionals are allowed to participate in the consultation of the ICU, with symptom control as the main content and with additional attention paid to spiritual care simultaneously. Nevertheless, the disadvantage of this consultation mode is that HPC professionals are insufficient to meet all the needs of HPC in the ICU. Moreover, excessive reliance on HPC consultation may imbalance the therapeutic relationship between doctors and patients in ICU, resulting in a fragmented medical mode. In addition, the implementation of HPC consultation will reduce the demand for doctors in ICU to learn PC skills and knowledge. HPC consultation can be initiated when the HPC concept has not been integrated into the knowledge system of doctors in the ICU.

Therefore, it is recommended currently to combine the integration mode with the consultation mode. It has been documented that the practice of the mixed mode can improve the quality of life of patients, improve the signing rate of ACP and the utilization rate of HPC institutions, and reduce the utilization rate of ineffective life maintenance treatment (10).

## 8. HPC skills required by doctors in ICU

To provide good services for HPC of critically ill patients, doctors in the ICU need to have the following abilities:

### 8.1. The ability to improve the professional ability of intensive care to accurately control the disease

Doctors of critical care medicine are required to have professional quality and ability. It not only contributes to the determination of the patient's condition and severity at the first time but also can make an appropriate and accurate judgment on the treatment response. Based on this ability, doctors can apply appropriate treatment according to patients' conditions, rather than “overtreatment.”

### 8.2. Ability to predict and fully inform

The doctors in ICU should have the ability to predict (e.g., painful symptoms, pain, thirst, anxiety, etc.) what the patients will experience during their stay in the ICU, the state of the patients after entering the ICU, the possible therapeutic effect, possible outcome, the possible cost of treatment in ICU, ways for family members to visit and accompany patients, post-ICU syndrome, and the experience and common reactions of family members such as psychological and physical pain, depression, and post-traumatic syndrome and fully communicate with the patients/families. The key lies in that in addition to communicating with the patients/families about “the necessity and possible benefits of treatment in ICU,” it is important to fully explain “the painful situation of patients in ICU, the proportion of poor prognosis, high costs, and many other unfavorable details.” Based on this, the patient can make the important decision of “whether to stay in the ICU or not.” In many cases, doctors in ICU are busy with their work in emergency situations and fail to inform the patients/families of the latter details.

### 8.3. Ability to hold family meetings and do shared decision-making

Family meetings are the work that doctors in ICU deal with every day. It is challenging for doctors to make better joint decisions at any time based on a family meeting. Among them, the communication contents and technical points involved generally in the family meetings of critically ill patients include not giving or withdrawing a certain treatment, available options at the end of life, the purpose of treatment, the methods to reduce pain, the wishes of patients, the arrangement of things after death, and the peace of mind of family members are the talking contents and technical points often involved in family meetings for severe patients.

### 8.4. Ability to communicate well with families

Both patients and their families prefer honest, respectful, and sympathetic communication with the feeling of being listened to. Previous research has revealed that the way the doctors in the ICU guide conversations would affect the therapeutic relationship between doctors and patients and the acceptance of their families (11). A planned communication framework is as important as the specific content of communication, e.g., being able to find out the key family members who can promote communication and the people who are more willing to discuss the end-of-life topics in the family of the patients, being able to detect and deal with the complex emotions of the patients/families, such as anxiety, fear, anger, entanglement, and reluctance, and being able to carry out discussion on the topics related to ethics clearly and methodically, i.e., respecting patients' autonomy, being beneficial, not harmful, patients' decision-making ability, and determining entrusted agents.

### 8.5. Ability to effectively deal with patients' physical, psychological, social, and spiritual pain

This ability involves non-drug pain relief methods and the use of analgesic and sedative drugs. The ability to pay attention to and care for psychological, social, and spiritual pain is also quite important in ICU, in addition to the ability to control the physical symptoms of the patients. It has been reported that dignity therapy, life review, personal narration, and wish fulfillment (12) are all available methods with significant effects. It is particularly worth emphasizing that the aforementioned contents need to be prepared and intervened as soon as possible. According to multiple clinical cases, it is time-consuming to prepare for death! It needs to be proposed early! It is not recommended to discuss this issue only a few hours or minutes before death.

### 8.6. Ability to self-learning

Currently, many resources and tools are available to assist doctors in critical care medicine to learn about primary PC (13).

## 9. Obstacles to the integration of critical care medicine and HPC

Obstacles to the integration of critical care medicine and HPC mainly include the following two parts: (1) it is mistakenly believed that the treatment in ICU and HPC are related in time and thought that HPC can be carried out after there is nothing to deal with in ICU, without recognizing the coexisting and complementary relationship between them; and (2) the medical team and family members have unrealistic expectations for treatment and worry that the implementation of HPC is to give up the patients or even hasten the death. All these views may lead to insufficient training of the medical team in HPC skills.

The aging of the population accelerates the occurrence of death. With the development of the social economy and the improvement of the level of civilization, there is a constant improvement in the public's requirements for quality of life, including the rigid demand for a good ending and peace between life and death. All medical staff should have the ability to help patients die well. In particular, the medical staff of critical care medicine bears a more important mission. It is critical to maintaining the balance between life and death, which embodies the difficulty and responsibility of the clinical practice of the medical staff in critical care medicine more significantly.

## 10. Prospective future

China's critical care medical treatment has just begun, and much data is still blank. The focus and core of our work lie in how to balance the boundaries of intensive care and palliative care. This requires intensivists to know the content of HPC in the ICU and master HPC skills and perform special practice in the daily work at the same time. In the future, China will accelerate into aging, and the role of HPC in the ICU is about to be highlighted. It is necessary to speed up training and improve the perception of employees.

## Author contributions

Both authors listed have made a substantial, direct, and intellectual contribution to the work and approved it for publication.
